# Beyond Carbapenemases: Chromosomal Resistance Mechanisms and Carbapenemase Type Determine Susceptibility to Novel β-Lactam/β-Lactamase Inhibitor Combinations and Cefiderocol in Carbapenem-Resistant *Pseudomonas aeruginosa* from China

**DOI:** 10.3390/antibiotics15090835

**Published:** 2026-08-27

**Authors:** Jiankang Zhao, Yanyan Fan, Bin Cao

**Affiliations:** 1 National Center for Respiratory Medicine, State Key Laboratory of Respiratory Health and Multimorbidity, New Cornerstone Science Laboratory, National Clinical Research Center for Respiratory Diseases, Institute of Respiratory Medicine, Chinese Academy of Medical Sciences, Department of Pulmonary and Critical Care Medicine, Center of Respiratory Medicine, China-Japan Friendship Hospital, Beijing 100029, China; zjk4265296@163.com (J.Z.); fanyanyan0302@126.com (Y.F.); 2 Department of Pulmonary and Critical Care Medicine, Capital Medical University, Beijing 100069, China

**Keywords:** carbapenem-resistant *Pseudomonas aeruginosa*, β-lactam/β-lactamase inhibitors, chromosomal resistance mechanisms, carbapenemase genes, cefiderocol

## Abstract

Background: β-lactam/β-lactamase inhibitor combinations (BL/BLIs) and siderophore cephalosporin serve as critical polymyxin alternatives for carbapenem-resistant *Pseudomonas aeruginosa* (CRPA). However, systematic multicenter clinical data from China and the resistance mechanisms underlying non-susceptibility to these agents remain poorly defined. Methods: We collected 442 CRPA isolates from 10 hospitals across eight Chinese provinces (2017–2025), determined minimum inhibitory concentrations (MICs) for cefiderocol (FDC), ceftazidime–avibactam (CZA), imipenem–relebactam (IMR), and ceftolozane–tazobactam (CT) through broth microdilution, and performed whole-genome sequencing. Resistance genes, virulence factors, and sequence types (STs) were identified. A chromosomal co-selection score (eight non-mobile resistance genes) was constructed. Multivariable regression and receiver operating characteristic (ROC) analysis identified predictors of non-susceptibility. Results: Carbapenemase genes were detected in only 33 isolates (7.5%): metallo β lactamase (MBL) genes in 28, *bla*_KPC-2_ in 2, and *bla*_GES 5/14_ in 3; the ST463 clone was absent. MBL producers were resistant to CZA, IMR, and CT (0–4% susceptible) but 100% susceptible to FDC. KPC 2 and GES producers were CZA-susceptible (100%) but IMR- and CT-resistant. Among 127 CZA non-susceptible isolates, 99 (78%) lacked carbapenemase genes; ST270 (from a single center) accounted for 64.6% of these. The chromosomal score independently predicted MIC (*ρ*: CT 0.64, FDC 0.59, IMR 0.50, CZA 0.25—largely attributable to ST270; all *p* < 0.001). ExoU positive isolates had lower MICs (FDC median 0.12 vs. 0.25 mg/L, *p* < 0.001). FDC remained active against 97.3% of all isolates and 100% of carbapenemase-positive ones. Genotype-based prediction yielded area under the ROC curve (AUC) values of 0.86 (CT) and 0.80 (CZA). Conclusions: Mechanisms other than acquired carbapenemases—consistent with chromosomal determinants—accounted for most non-susceptibility in this predominantly northern Chinese respiratory tract CRPA collection. Carbapenemase types determine distinct drug susceptibility patterns. Routine carbapenemase detection fails to identify most CZA-resistant strains. FDC retained the broadest in vitro activity and is a reliable therapeutic option pending clinical outcome data, and ST270-associated resistance deserves continuous surveillance.

## 1. Introduction

Carbapenem-resistant *Pseudomonas aeruginosa* (CRPA) is designated a high-priority pathogen in the WHO Bacterial Priority Pathogens List 2024, reflecting its substantial disease burden and limited therapeutic options [1]. In the global prospective observational cohort of carbapenem-resistant *P. aeruginosa* (POP; 972 patients, 10 countries), carbapenemase-producing CRPA (60% of cases) was associated with a 7% absolute increase in 30-day mortality versus non-carbapenemase-producing strains [2]. In China, a multicenter retrospective study of 285 *P. aeruginosa* bloodstream infections reported a 34% carbapenem resistance rate, with inappropriate definitive therapy as the strongest independent predictor of 30-day mortality (adjusted OR 4.47) [3]. The resistance architecture of *P. aeruginosa* is uniquely multifaceted: resistance may arise from horizontally acquired carbapenemase genes, chromosomal mutations derepressing intrinsic AmpC β-lactamase, OprD porin loss/inactivation, and efflux pump overexpression—often coexisting in a single isolate [4,5,6].

The therapeutic landscape for CRPA has expanded notably with novel β-lactam/β-lactamase inhibitor (BL/BLI) combinations and the siderophore cephalosporin cefiderocol (FDC). Ceftazidime–avibactam (CZA) and imipenem–relebactam (IMR) rely on diazabicyclooctane inhibitors (avibactam and relebactam, respectively) that inactivate class A and C β-lactamases but not metallo-β-lactamases (MBLs); IMR activity is additionally compromised by OprD loss [4,7]. Ceftolozane–tazobactam (CT) couples a modified antipseudomonal cephalosporin—stable against AmpC and with enhanced penicillin-binding protein affinity—with tazobactam [4]. FDC employs a distinct Trojan horse mechanism: its catechol moiety chelates ferric iron, enabling active transport through bacterial siderophore uptake systems, thereby bypassing porin loss and efflux-mediated resistance while retaining stability against all four Amble β-lactamase classes [6]. The SENTRY program (2020–2023, 9572 *P. aeruginosa* isolates) quantified the clinical divergence: CZA (96.1%), IMR (96.5%), and CT (95.9%) showed >95% susceptibility in unselected isolates, but their activity fell to 52.3%, 56.0%, and 54.4%, respectively, against difficult-to-treat resistance (DTR) subsets. In contrast, FDC retained 98.1% susceptibility against DTR strains and 97.3% against carbapenemase-producing DTR isolates—a population for which all three BL/BLIs exhibited <6% activity [8]. BL/BLI activity is therefore mechanism-dependent and predictable only when the underlying resistance genotype is known [7,9].

The molecular epidemiology of CRPA in China carries direct implications for empiric therapy. Carbapenemase genes are detected in a minority of CRPA isolates nationwide; chromosomally mediated mechanisms—AmpC deregulation, OprD inactivation, and efflux overexpression—account for the majority of carbapenem-resistant phenotypes [4,5,10,11]. Among acquired carbapenemases, MBLs of the IMP and VIM families predominate. Sequence type 463 (ST463) has emerged as a dominant high-risk clone in eastern China. In a national collection of 1168 CRPA isolates, ST463 comprised 9.8% of strains with 81.7% IMR non-susceptibility despite KPC-2 being a class A serine carbapenemase ordinarily inhibited by relebactam; resistance was driven by elevated *bla*_KPC-2_ copy number and expression [11], while 93.96% of China clade ST463 isolates carry *bla*_KPC-2_ and all co-express ExoU and ExoS, key type III secretion system (T3SS) virulence effectors of *P. aeruginosa* [12]. ExoU mediates acute cytotoxicity, whereas ExoS interferes with host cellular functions to evade phagocytosis. Novel KPC-2 variants (KPC-14, KPC-33, KPC-86) conferring high-level CZA resistance have emerged within this lineage [13], alongside novel MBL variants, including VIM-92 in northern China [14] and AFM-type carbapenemases co-harbored with KPC-2 in central China [15]. However, because carbapenemase genes are absent in the majority of Chinese CRPA, chromosomally encoded mechanisms likely underpin most BL/BLI non-susceptibility, yet their contribution remains incompletely quantified [16].

This knowledge gap has direct clinical implications: current molecular diagnostics target only a defined panel of carbapenemase genes and cannot identify chromosomal resistance mechanisms [17]. No Chinese multicenter study has yet compared all four novel agents (FDC, CZA, IMR, CT) with comprehensive genomic characterization. To address this, we collected 442 CRPA isolates from 10 hospitals across eight provinces (2017–2025), determined MICs for the four drugs, performed whole-genome sequencing, and analyzed genotype–phenotype relationships. We found that carbapenemase genes were present in only 7.5% of isolates, the ST463 clone was absent, and a single chromosomal clone (ST270) accounted for the majority of CZA-non-susceptible, carbapenemase-negative strains—highlighting the need to recalibrate diagnostic approaches to guide therapy with novel agents.

## 2. Results

### 2.1. Resistance Gene Landscape of CRPA in China

#### 2.1.1. Study Population

Most isolates came from Beijing (*n* = 382, 86.4%, three hospitals), followed by Guangdong (*n* = 27, 6.1%), Jiangxi (*n* = 13, 2.9%), and Hebei (*n* = 7, 1.6%). All specimens were respiratory: sputum accounted for 231 isolates (52.3%) and bronchoalveolar lavage fluid (BALF) 211 (47.7%). Male patients contributed 336 isolates (76.0%). The median patient age was 67 years (range, 0.24–105 years).

#### 2.1.2. The Resistance Gene Repertoire

**Figure 1:**
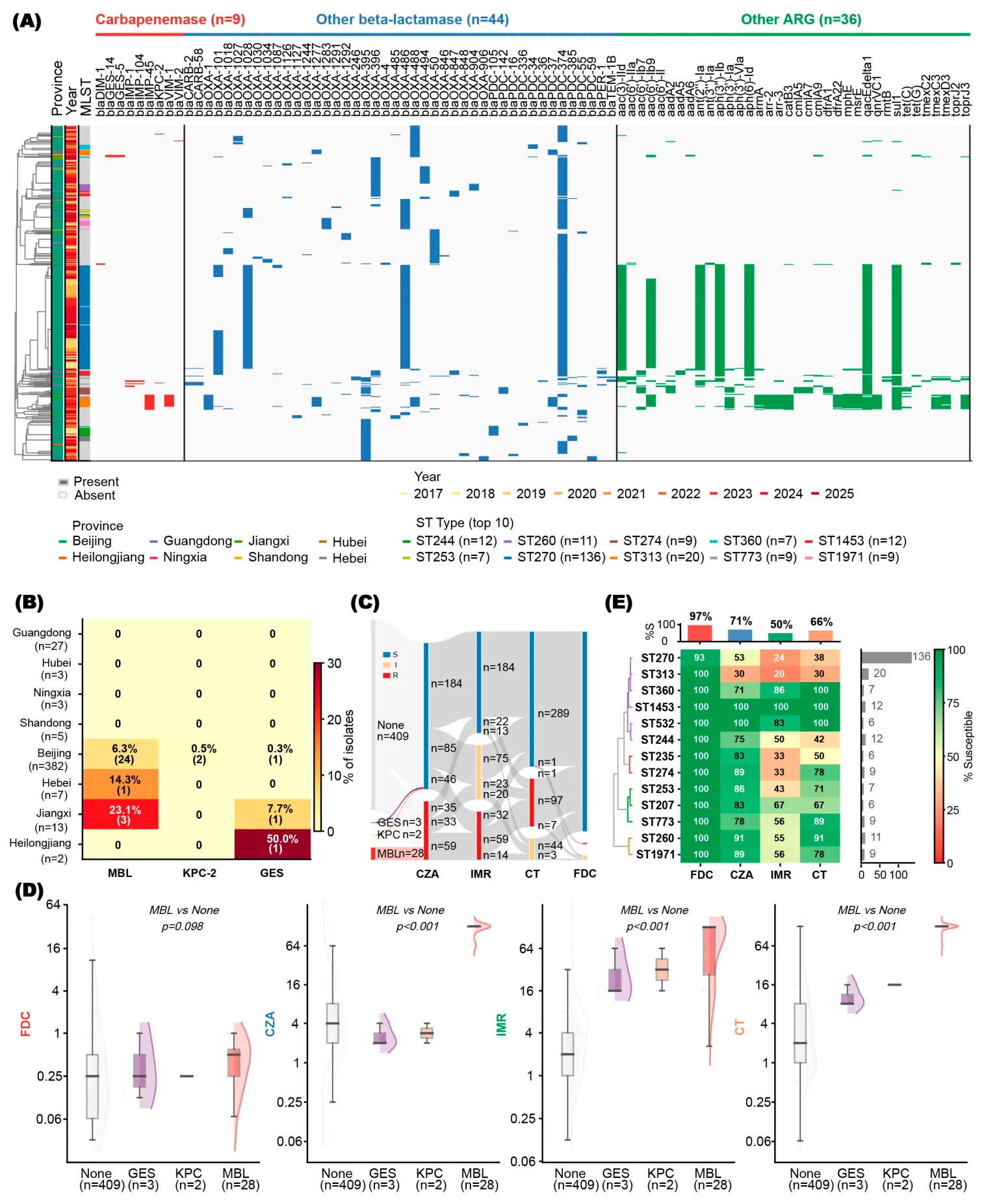
Resistance gene landscape and drug susceptibility patterns of 442 carbapenem-resistant *P. aeruginosa* isolates from China. (**A**) Hierarchically clustered heatmap of 89 resistance genes grouped into three functional categories (carbapenemase, *n* = 9; other β-lactamase, *n* = 44; other resistance genes, *n* = 36). Each row represents one isolate; each column represents one resistance gene. Red, blue, and green indicate gene presence in the respective category. Row sidebars show province, year of isolation, and sequence type. Strains were ordered by Jaccard distance (average linkage); genes were sorted alphabetically within each category. (**B**) Province-level distribution of carbapenemase types (MBL, KPC, GES) among the 33 carbapenemase-positive isolates. Cell color intensity indicates the percentage of isolates within each province carrying the indicated enzyme type. (**C**) Alluvial diagram illustrating the flow of isolates from carbapenemase type to the susceptible (S), intermediate (I), and resistant (R) categories for cefiderocol (FDC), ceftazidime–avibactam (CZA), imipenem–relebactam (IMR), and ceftolozane–tazobactam (CT). Block heights are proportional to the number of isolates; colors indicate carbapenemase type or S/I/R status. (**D**) Raincloud plots of MIC distributions for the four agents stratified by carbapenemase type (none, GES, KPC, MBL). Each panel shows individual data points (jittered), box plots (median, interquartile range, whiskers), and half-violin kernel density estimates. *p*-values are from Mann–Whitney *U* tests comparing MBL versus carbapenemase-negative groups. (**E**) Hierarchically clustered heatmap of percent susceptibility (%S) for the four agents across 13 sequence types (STs) with six or more isolates. Cells are colored from red (0% susceptible) to yellow and green (100% susceptible). The dendrogram (left) was derived from Ward’s method on Euclidean distances of %S vectors. Cluster brackets indicate the four major ST clusters. The bar chart (right) shows the number of isolates per ST.

We detected 221 unique resistance determinants, while 51 species-core determinants (>95% prevalence) were excluded from further analysis (examples include *mexAB*-*oprM* efflux pump components, intrinsic AmpC β-lactamase *bla*_PAO_, *aph(3′)-IIb*, and *fosA*). After excluding 81 rare genes (<3 strains, non-carbapenemase) and retaining nine carbapenemase genes regardless of prevalence, 89 genes were sorted into three functional categories: carbapenemase genes (*n* = 9), other β-lactamase genes (*n* = 44), and other resistance genes (*n* = 36). This filtering strategy was adopted to improve the interpretability of the heatmap; the full inventory of all 221 resistance determinants is provided in Supplementary Table S1. Notably, the heatmap only illustrates the presence or absence of each resistance gene and does not account for sequence variants/mutations within these genes. Figure 1A shows these 89 genes as a hierarchically clustered heatmap (strains ordered by Jaccard distance and average linkage; genes sorted alphabetically within category).

The nine carbapenemase genes were *bla*_IMP-45_ (*n* = 20, 4.5%), *bla*_VIM-1_ (*n* = 16, 3.6%), *bla*_IMP-1_ (*n* = 5, 1.1%), *bla*_GES-5_ (*n* = 3, 0.7%), *bla*_GES-14_ (*n* = 3, 0.7%), *bla*_IMP-104_ (*n* = 3, 0.7%), *bla*_KPC-2_ (*n* = 2, 0.5%), *bla*_DIM-1_ (*n* = 2, 0.5%), and *bla*_VIM-2_ (*n* = 1, 0.2%). Altogether, 33 out of 442 strains (7.5%) carried at least one carbapenemase gene. Of these, 28 strains carried MBL genes (IMP, VIM, or DIM families). Two strains carried *bla*_KPC-2_, and three strains co-harbored *bla*_GES-5_ and *bla*_GES-14_. Co-carriage of multiple MBL genes was common: 16 out of 20 IMP-45-positive strains also carried VIM-1, and three IMP-1-positive strains also carried IMP-104.

The other β-lactamase category (*n* = 44) was dominated by intrinsic OXA-50-family variants (*bla*_OXA-486_, 40.7%; *bla*_OXA-488_, 12.9%; *bla*_OXA-101_, 17.4%). It also contained acquired narrow-spectrum β-lactamases such as *bla*_PER-1_ (2.3%) and *bla*_OXA-1_ (4.5%). The other resistance gene category (*n* = 36) consisted mainly of aminoglycoside-modifying enzymes: *aph(3″)-Ib* (35.1%), *aph(6)-Id* (34.6%), *aac(3)-IId* (33.9%), *ant(2″)-Ia* (33.9%), and *aac(6′)-Ib9* (31.4%). *sul1* was the most prevalent non-β-lactamase gene (42.8%).

#### 2.1.3. Geographic Distribution of Carbapenemase Genes

Carbapenemase gene carriage varied significantly across the eight provinces (chi-square test, *p* = 0.008). Figure 1B shows the province-level distribution. Jiangxi had the highest positivity rate (30.8%, 4 of 13 strains; three co-carried IMP-45 and VIM-1; one carried GES-5 and GES-14). Heilongjiang had one GES-5/GES-14-positive strain among its two isolates. Hebei had one DIM-1-positive strain among seven isolates. Beijing contributed 86% of all isolates but had a carbapenemase positivity rate of only 7.1% (27 of 382) while showing the greatest diversity of carbapenemase types. Four provinces had no carbapenemase-positive strains: Guangdong (*n* = 27), Shandong (*n* = 5), Ningxia (*n* = 3), and Hubei (*n* = 3).

#### 2.1.4. Carbapenemase Type Predicts Drug-Specific Susceptibility

Carbapenemase type determined the pattern of susceptibility to each agent. Figure 1C shows the alluvial flow of strains from carbapenemase type to the susceptible/intermediate/resistant (S/I/R) categories for each drug. Observed susceptibility rates by carbapenemase type were as follows. MBL producers (*n* = 28): FDC 100% S, CZA 0% S, IMR 4% S (1 of 28), CT 0% S. KPC-2 producers (*n* = 2): FDC 100% S, CZA 100% S, IMR 0% S, CT 0% S. GES-5/14 producers (*n* = 3): FDC 100% S, CZA 100% S, IMR 0% S, CT 0% S.

The MIC distributions were displayed as raincloud plots (Figure 1D). FDC MICs did not differ significantly between MBL-producing and carbapenemase-negative strains (Mann–Whitney *U* test, *p* = 0.098; median MIC: MBL 0.50 mg/L, carbapenemase-negative 0.25 mg/L). All 33 carbapenemase-positive strains were FDC-susceptible. CZA MICs were significantly higher in MBL producers (median > 64 vs. 4 mg/L, *p* < 0.001). KPC-2 and GES-5/14 producers remained CZA-susceptible (MIC range, 2–4 mg/L). IMR MICs were also elevated in MBL producers (median > 64 vs. 2 mg/L, *p* < 0.001); one IMP-1-positive strain was an exception (MIC 1 mg/L). KPC-2 producers were IMR-resistant (MIC 16–64 mg/L), consistent with OprD porin deficiency, which impairs IMR activity independently of serine carbapenemase, and/or with increased *bla*_KPC-2_ copy number and expression overwhelming the relebactam, as reported for ST463 [11]; neither mechanism was directly measured in these two isolates. GES-5/14 producers were IMR-resistant or intermediate (MIC 8–16 mg/L). CT MICs were higher in MBL producers (median > 64 vs. 2 mg/L; *p* < 0.001); all MBL, KPC-2, and GES-5/14 producers were CT non-susceptible (MIC 8–>64 mg/L).

#### 2.1.5. Clonal Structure of Susceptibility

We identified 136 sequence types (STs) among the 425 typeable isolates, while 17 strains could not be assigned an ST. Thirteen STs contained six or more isolates and accounted for 250 out of 442 strains (56.6%). Figure 1E shows the ST–drug susceptibility landscape as a hierarchically clustered heatmap (Ward’s method on the Euclidean distance of percent-susceptible vectors).

Four clusters emerged. Cluster 1 contained ST270 (*n* = 136, 30.8%), ST313 (*n* = 20), ST244 (*n* = 12), ST1453 (*n* = 12), ST360 (*n* = 7), and ST532 (*n* = 6). ST270 came almost entirely from Beijing (134 out of 136 strains) and had the lowest susceptibility among major clones (FDC-S 92.6%, CZA-S 52.9%, IMR-S 24.3%, CT-S 37.5%.); no ST270 strain carried a carbapenemase gene. ST313 was the dominant IMP-45/VIM-1-carrying clone. ST1453, ST532, and ST360 showed high susceptibility (≥71% susceptibility to all drugs; no carbapenemase genes). Cluster 2 comprised ST235 (*n* = 6) and ST274 (*n* = 9). Cluster 3 included ST253 (*n* = 7), ST207 (*n* = 6), and ST773 (*n* = 9). Cluster 4 contained ST260 (*n* = 11) and ST1971 (*n* = 9). ST235, a globally recognized high-risk clone, showed IMR-S 33.3% and CT-S 50.0%. ST463, recently reported as a dominant KPC-2-producing high-risk clone in eastern China, was absent from this collection.

### 2.2. CZA Non-Susceptibility

#### 2.2.1. Magnitude of the Gap

**Figure 2:**
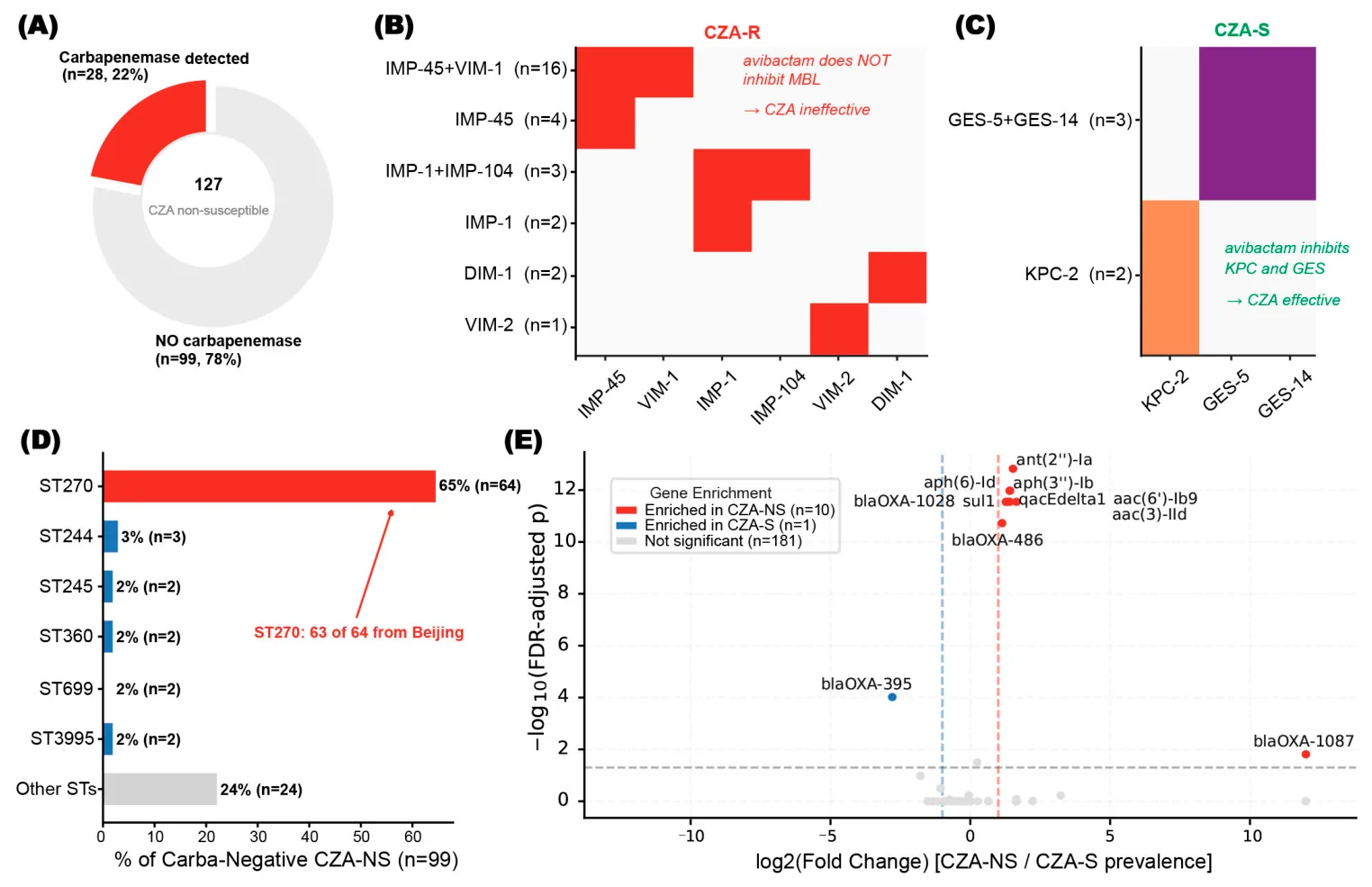
The carbapenemase detection gap in ceftazidime–avibactam non-susceptibility. (**A**) Donut chart showing the proportion of CZA-non-susceptible isolates (*n* = 127) with a detectable carbapenemase gene (red, *n* = 28, 22%) versus those without (gray, *n* = 99, 78%). (**B**) Binary heatmap of unique metallo-β-lactamase (MBL) gene combinations among the 28 CZA-resistant, carbapenemase-positive isolates. Red indicates gene presence; white indicates absence. Row labels show the number of isolates with each gene profile. (**C**) Heatmap of carbapenemase gene combinations among the five CZA-susceptible, carbapenemase-positive isolates. Orange (KPC-2) and purple (GES-5/GES-14) indicate gene presence. (**D**) Horizontal bar chart showing the clonal distribution of CZA-non-susceptible, carbapenemase-negative isolates (*n* = 99). ST270 accounted for 64.6% (63 from Beijing, 1 from Guangdong). (**E**) Volcano plot of all 221 resistance determinants screened for enrichment in CZA-non-susceptible versus CZA-susceptible strains within the carbapenemase-negative subset (*n* = 409). The *x*-axis shows log2 fold change in gene prevalence; the *y*-axis shows −log10(*p*-value) from Fisher’s exact test. Red points: significantly enriched in CZA-non-susceptible isolates (fold change > 2, *p* < 0.05). Blue points: enriched in CZA-susceptible isolates. Gray: not significant. Selected genes are labeled.

Among the 127 CZA non-susceptible strains, only 28 (22.0%) carried a detectable carbapenemase gene. The other 99 strains (78.0%) had no known carbapenemase gene. Carbapenemase-centered molecular testing would therefore miss the majority of CZA resistance. Figure 2A shows this gap as a donut chart.

#### 2.2.2. Carbapenemase Type Governs CZA Susceptibility

All 28 CZA non-susceptible, carbapenemase-positive strains carried MBL genes (MBLs are not inhibited by avibactam). IMP-45 and VIM-1 co-carriage was the most common profile (16 of 28, 57.1%). Five carbapenemase-positive strains were CZA-susceptible: two carried *bla*_KPC-2_ alone (ST377, ST1295, Beijing) and three co-carried *bla*_GES-5_ and *bla*_GES-14_ (ST235 and ST253 from Heilongjiang, Jiangxi, and Beijing), consistent with avibactam inhibition of serine β-lactamases (Figure 2B,C).

#### 2.2.3. Clonal Source and Molecular Correlates

Among the 99 carbapenemase-negative CZA non-susceptible strains, ST270 accounted for 64 strains (64.6%), out of which 63 were from Beijing and 1 was from Guangdong (Figure 2D). The remaining 35 strains were distributed across 25 STs. A genome-wide screen of all 221 resistance determinants in the carbapenemase-negative subset (Figure 2E) identified 10 genes significantly enriched in CZA-non-susceptible strains (fold change [FC] > 2, Benjamini–Hochberg false discovery rate [FDR]-adjusted *p* < 0.05). Aminoglycoside-modifying enzyme genes were the most robust and prevalent signals: *aac(3)-IId* (68% in CZA-NS vs. 25% in CZA-S; FC 2.7; *p* < 0.001), *ant(2″)-Ia* (70% vs. 24%; FC 2.9), *aph(3″)-Ib* (70% vs. 26%; FC 2.7), *aph(6)-Id* (70% vs. 26%; FC 2.7), *sul1* (73% vs. 30%; FC 2.4), *qacEdelta1* (73% vs. 30%; FC 2.4), *aac(6′)-Ib9* (60% vs. 19%; FC 3.1), and three OXA-family β-lactamase variants (*bla*_OXA-1028_, 69% vs. 26%; *bla*_OXA-486_, 76% vs. 35%; *bla*_OXA-1087_, 5% vs. 0%). No carbapenemase or extended-spectrum β-lactamase gene reached significance.

### 2.3. Cefiderocol Evades Known Resistance Mechanisms

#### 2.3.1. FDC Activity Across Genotypes

**Figure 3:**
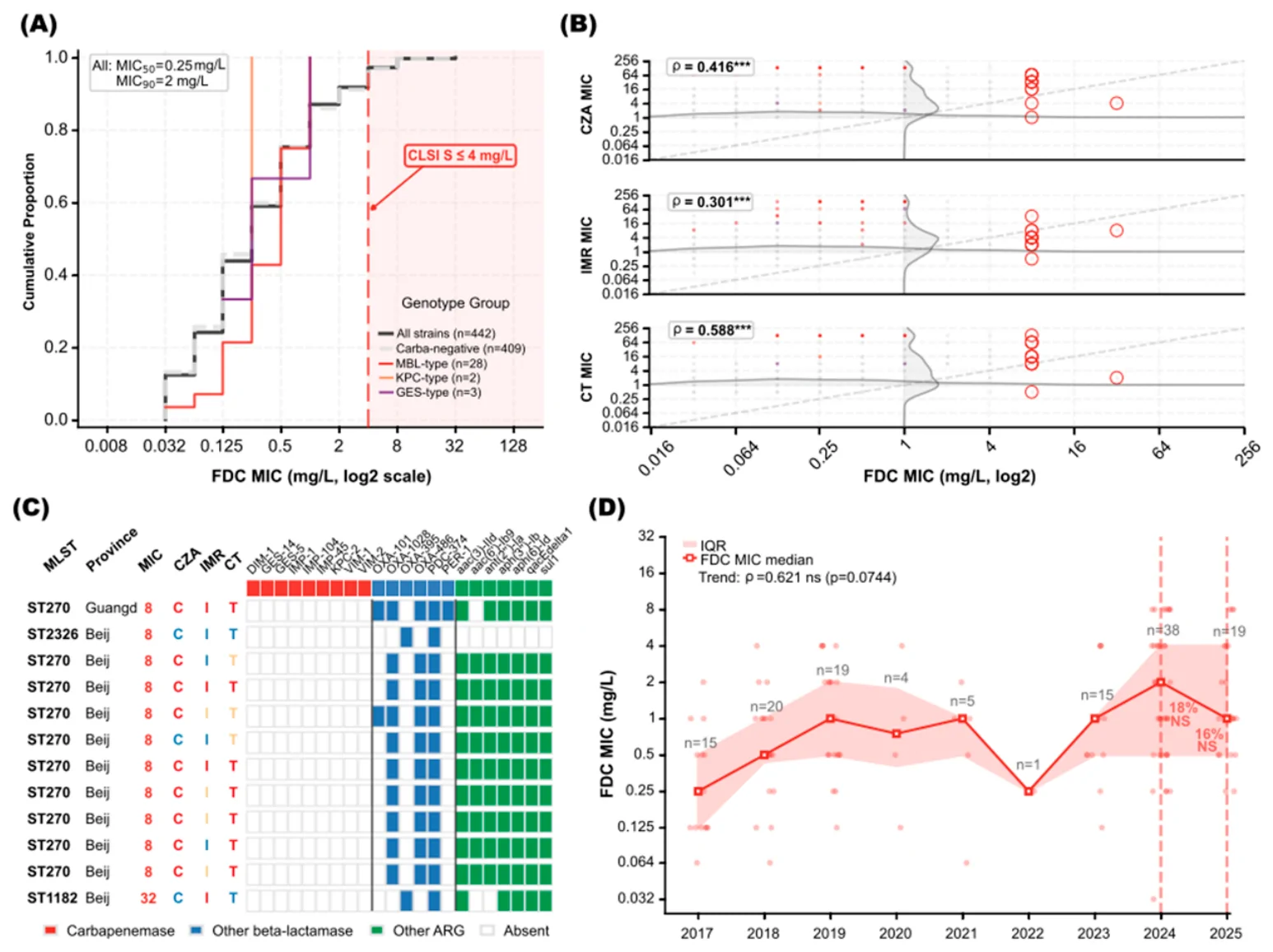
Cefiderocol activity and molecular features of non-susceptible isolates. (**A**) Empirical cumulative distribution functions (ECDFs) of FDC MIC stratified by carbapenemase type. The vertical dashed line marks the CLSI 2025 susceptible breakpoint (≤4 mg/L); the red shaded area indicates the non-susceptible range (>4 mg/L). (**B**) Log2–log2 scatter plots of FDC MIC versus CZA, IMR, and CT MIC. Points are colored by carbapenemase type. Large red open circles indicate FDC-non-susceptible isolates (*n* = 12). The dashed diagonal line represents y = x. Marginal density curves show the distribution of MICs for each drug. Spearman correlation coefficients (*ρ*) and significance are shown in the upper left of each panel. *** indicates *p* < 0.001. (**C**) Gene by gene molecular portrait of the 12 FDC-non-susceptible isolates. Genes were selected for display if they were carbapenemase genes (included regardless of prevalence), had two or more occurrences among the 12 isolates, or carried established biological relevance (*bla*_PER-1_). Red, blue, and green indicate gene presence in the respective functional category. Metadata columns show ST, province of origin, FDC MIC, and susceptibility status for CZA, IMR, and CT. (**D**) Temporal trajectory of FDC MIC among ST270 isolates (*n* = 136) from 2017 to 2025. Circles represent individual isolates; the solid line and shaded ribbon show the median and interquartile range per year. Vertical dashed lines mark the years in which FDC-non-susceptible ST270 isolates appeared. The Spearman rank correlation (*ρ*) and *p*-value test were used for the temporal trend.

FDC retained potent in vitro activity across all molecular subgroups. The five empirical cumulative distribution function (ECDF) curves (all strains, carbapenemase-negative, MBL, KPC, GES) were nearly superimposable (Figure 3A). The overall FDC MIC_50_ was 0.25 mg/L. The CLSI 2025 susceptible breakpoint (≤4 mg/L) captured 97.3% (430 of 442) out of all strains and 100% (33 of 33) of carbapenemase-positive strains. Only 12 strains (2.7%) were FDC non-susceptible (11 intermediate, 1 resistant); all were carbapenemase-negative.

#### 2.3.2. FDC MIC Does Not Track BL/BLI MICs

Figure 3B shows three log_2_–log_2_ scatter plots of FDC MIC against CZA, IMR, and CT MIC. The Spearman correlation between FDC and CZA MIC was 0.416, between FDC and IMR MIC was 0.301, and between FDC and CT MIC was 0.588 (all *p* < 0.001). These moderate correlations underscore that FDC resistance is mechanistically distinct from resistance to the three BL/BLIs.

#### 2.3.3. Molecular Portrait of FDC Non-Susceptible Strains

A gene by gene view of the 12 FDC non-susceptible strains is shown in Figure 3C. All nine carbapenemase genes were absent. The AmpC variant *bla*_PDC-374_ was present in all 12 strains. Aminoglycoside-modifying enzyme genes were present in nearly all strains, and *sul1*/*qacEdelta1* were each present in 11 strains. Ten strains belonged to ST270 (nine from Beijing, one from Guangdong); all ten had FDC MICs of 8 mg/L (intermediate).

#### 2.3.4. FDC Non-Susceptibility Emerged over Time in ST270

The first FDC non-susceptible strain was isolated in 2019 (ST2326, FDC MIC 8 mg/L, Beijing), followed by a second strain in 2022 (ST1182, FDC MIC 32 mg/L, Beijing). Neither belonged to ST270. Figure 3D tracks the FDC MIC of ST270 from 2017 to 2025. No FDC non-susceptible ST270 strain was detected before 2024. Seven out of the ten appeared in 2024, and the remaining three appeared in 2025. The median FDC MIC in ST270 rose modestly (Spearman *ρ* = 0.22, *p* = 0.009). By 2025, the cumulative proportion of FDC non-susceptible strains among ST270 reached 7.4% (10 of 136). ST270 did not acquire any carbapenemase genes during this period; the MIC increase may be driven by chromosomal mechanisms, although the cross-sectional design precludes causal inference.

### 2.4. Cross-Resistance and Non-Carbapenemase Co-Selection

#### 2.4.1. Molecular Makeup of Cross-Resistance Phenotypes

**Figure 4:**
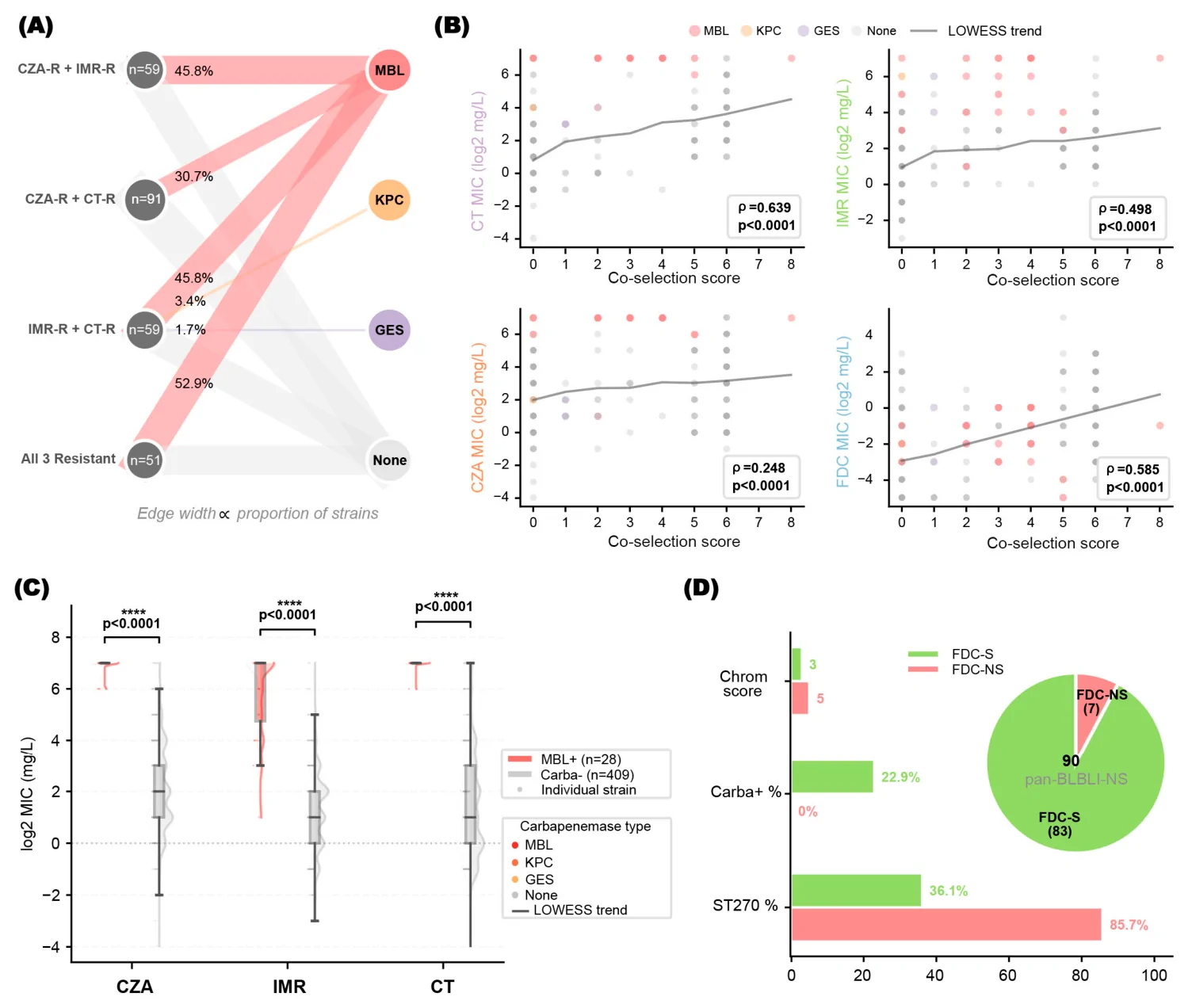
Cross-resistance networks, non-carbapenemase co-selection, and pan-BL/BLI-non-susceptible isolates. (**A**) Bipartite network connecting cross-resistance phenotypes (left nodes; gray circles) to carbapenemase types (right nodes; colored circles). Edge width is proportional to the fraction of isolates within the phenotype attributable to the indicated carbapenemase type. Node sizes reflect the total number of isolates. (**B**) Non-carbapenemase co-selection score (range 0–8) versus log2 MIC for CT, IMR, CZA, and FDC. Points are colored by carbapenemase type. The black line is a LOWESS trend line. Spearman *ρ* and *p*-values are shown in the lower right of each panel. (**C**) Raincloud plots comparing MIC distributions between carbapenemase-negative isolates (gray; chromosomal resistance only, *n* = 409) and MBL-producing isolates (red; *n* = 28) for CZA, IMR, and CT. Half-violins show kernel density estimates; boxes show median and interquartile range. *p*-values are from Mann–Whitney *U* tests with significance brackets. *n* values are shown below each group. **** indicates *p* < 0.0001. (**D**) Comparison of FDC-susceptible (*n* = 83; green) and FDC-non-susceptible (*n* = 7; red) subgroups among the 90 pan-BL/BLI-non-susceptible (CZA-NS + IMR-NS + CT-NS) isolates. Left: donut chart of the 90 pan-BL/BLI-NS isolates. Right: grouped horizontal bars comparing the percentage of ST270, the percentage of carbapenemase-positive isolates, and the median non-carbapenemase co-selection score between the two subgroups.

Among the 442 strains, 90 (20.4%) were non-susceptible to CZA, IMR, and CT simultaneously (pan-BL/BLI-NS), while 51 (11.5%) were resistant to all three drugs. MBL producers were heavily concentrated in the most severe cross-resistance categories: 52.9% (27 of 51) of all-three-resistant strains and 30.0% (27 of 90) of pan-BL/BLI-NS strains. Most cross-resistance strains carried no carbapenemase genes, particularly in CZA-R plus CT-R (69.2% carbapenemase-negative) and pan-BL/BLI-NS (70.0% carbapenemase-negative) (Figure 4A).

#### 2.4.2. Non-Carbapenemase Co-Selection Score and MIC

Eight non-carbapenemase resistance genes with 5–95% prevalence constituted the co-selection score (range 0–8; see Section 4). The median score was 0 (IQR, 0–6) and 244 strains (55.2%) had a score of 0, while 159 (36.0%) had a score ≥ 4 (Figure 4B).

The score correlated with MIC in a drug-dependent manner. For CT, the correlation was strongest (Spearman *ρ* = 0.639, *p* < 0.001). Among carbapenemase-negative strains with a score of 0, median CT MIC was 1.0 mg/L, and 11.3% were CT non-susceptible; at ≥4, median CT MIC was 8.0 mg/L and 61.5% were CT non-susceptible (*p* < 0.001). For IMR, correlation was moderate (*ρ* = 0.498). For FDC, correlation was *ρ* = 0.586 but driven largely by ST270 (among non-ST270 strains, *ρ* fell to 0.273; median FDC MIC among non-ST270, carbapenemase-negative strains remained 0.125 mg/L regardless of score). This attenuation indicates substantial clonal confounding, and the FDC association should be interpreted accordingly. For CZA, the correlation was weakest (*ρ* = 0.248). MBL-producing strains (*n* = 28) had uniformly elevated MICs for CZA, IMR, and CT irrespective of chromosomal score.

#### 2.4.3. Chromosomal vs. MBL-Mediated Resistance

MBL producers had substantially higher MICs than carbapenemase-negative strains for CZA (>64 vs. 4 mg/L, >16-fold), IMR (>64 vs. 2 mg/L, >32-fold), and CT (>64 vs. 2 mg/L, >32-fold) (Mann–Whitney *p* < 0.001 for each; Figure 4C). Chromosomal mechanisms raise MICs, but the effect is far smaller than that of an acquired MBL gene.

#### 2.4.4. Pan-BL/BLI Non-Susceptible Strains: FDC-Susceptible vs. FDC Non-Susceptible

Among 90 pan-BL/BLI-NS strains, 83 (92.2%) remained FDC-susceptible and 7 (7.8%) were FDC non-susceptible (Figure 4D). Carbapenemase genes were detected in 19 out of 90 (21.1%), all in the FDC-susceptible subgroup. The FDC non-susceptible subgroup was enriched for ST270 (6 of 7 vs. 30 of 83), had a higher median chromosomal score (5 vs. 3, *p* = 0.03), and showed higher CZA MIC (median 8.0 vs. 4.0 mg/L).

### 2.5. Virulence–Resistance Association and Clinical Translation

#### 2.5.1. Distribution of T3SS Effector Genes

**Figure 5:**
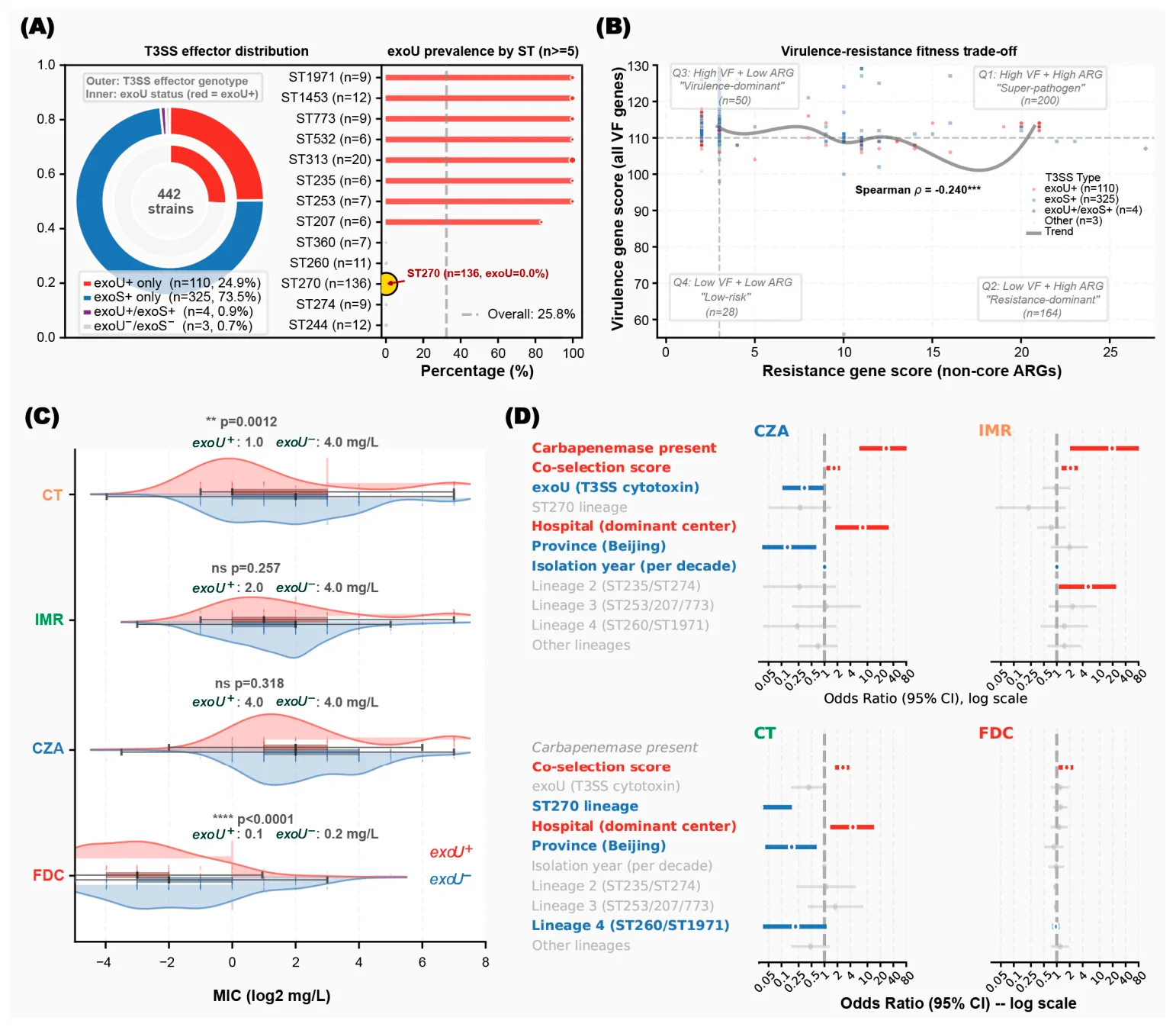
Virulence–resistance association, independent predictors of non-susceptibility, and genotype-based prediction. (**A**) Nested donut chart of T3SS effector genotypes. Outer ring: combined exoU/exoS genotype (cytotoxic *exoU*^+^/*exoS*^−^, invasive *exoU*^−^/*exoS*^+^, dual-positive *exoU*^+^/*exoS*^+^, dual-negative). Inner ring: *exoU* status alone. Right panel: horizontal lollipop chart of *exoU* carriage rates across STs with six or more isolates. (**B**) Virulence gene score (sum of 136 VFDB-detected virulence factors) plotted against resistance gene score (sum of non-core resistance determinants). Points are colored by T3SS effector genotype. Quadrant lines are drawn at the medians of each score (VF = 110, ARG = 3). The Spearman correlation coefficient (*ρ*) and *p*-value are shown. (**C**) Raincloud plots comparing MIC distributions between *exoU*-positive (*n* = 114; red) and *exoU*-negative (*n* = 328; blue) isolates for FDC, CZA, IMR, and CT. *p*-values from Mann–Whitney *U* tests and median MICs are annotated for each drug. (**D**) Forest plot of adjusted odds ratios (OR) and 95% confidence intervals from multivariable logistic regression for CZA, IMR, and CT non-susceptibility. Predictors shown are carbapenemase carriage, non-carbapenemase co-selection score, *exoU* positivity, ST270 membership, age (per 15 years), and male gender. Red circles indicate OR > 1 (risk factor); blue circles indicate OR < 1 (protective). Filled circles: *p* < 0.05. ** indicates *p* < 0.01, *** indicates *p* < 0.001, **** indicates *p* < 0.0001.

The T3SS effector genotype was determined for all 442 strains (Figure 5A). Figure 5A shows the distribution as a nested donut chart. The cytotoxic type (*exoU*^+^/*exoS*^−^) accounted for 24.9% (110 of 442); the invasive type (*exoU*^−^/*exoS*^+^) for 73.5% (325 of 442); dual-positive (*exoU*^+^/*exoS*^+^) for 0.9% (4 of 442). Three strains (0.7%) were dual-negative. *exoU* carriage varied widely across STs: all strains of ST532, ST1453, ST313, and ST253 were *exoU*-positive, whereas ST360 (0 out of 7) and ST270 (0 out of 136) were uniformly *exoU*-negative.

#### 2.5.2. Negative Association Between Virulence and Resistance Gene Burdens

A genome-wide virulence gene score (sum of 136 VFDB-detected factors) and a resistance gene score (sum of non-core resistance determinants) were negatively correlated (Spearman *ρ* = −0.240, *p* < 0.001; Figure 5B). The high-ARG/low-VF quadrant contained 164 strains (37.1%), including 113 out of 136 ST270 strains (83.1%). *exoU*^+^ strains were underrepresented in the resistance-dominant quadrant (9.6%, 11 of 114) compared with *exoU*^−^ strains (46.6%, 153 of 328; *p* < 0.001).

#### 2.5.3. Cytotoxic Strains Are More Usceptible

*exoU*^+^ strains had lower MICs for FDC (median 0.12 vs. 0.25 mg/L; *p* < 0.001) and CT (median 1.0 vs. 4.0 mg/L; *p* < 0.001) (Figure 5C). Differences were not significant for IMR (*p* = 0.257) or CZA (*p* = 0.318). The most cytotoxic strains were paradoxically the most susceptible to novel agents.

#### 2.5.4. Independent Predictors of Non-Susceptibility

Multivariable logistic regression identified independent predictors of non-susceptibility (Figure 5D). For CZA, carbapenemase carriage was the strongest predictor (adjusted OR 26.3, 95% CI 7.3–94.6, *p* < 0.001), followed by co-selection score (OR 1.60 per unit, 95% CI 1.25–2.05, *p* < 0.001); ExoU positivity was independently protective (OR 0.32, 95% CI 0.12–0.86, *p* = 0.023). For IMR, carbapenemase carriage (OR 18.7, 95% CI 2.3–151.9, *p* = 0.006), co-selection score (OR 2.00, 95% CI 1.45–2.75, *p* < 0.001), and lineage 2 membership (ST235/ST274; OR 5.17, 95% CI 1.26–21.2, *p* = 0.023) were significant. For CT, carbapenemase carriage showed near-perfect separation (all 33 carbapenemase-positive isolates were CT non-susceptible) and was excluded from the model; co-selection score (OR 2.58, 95% CI 1.98–3.36, *p* < 0.001) and lineage 4 membership (ST260/ST1971; OR 0.20, *p* = 0.049) were significant. For FDC, the unregularized primary model did not converge owing to only 12 non-susceptible isolates; in a regularized sensitivity model, the co-selection score (OR 1.63, 95% CI 1.24–2.14, *p* < 0.001) was associated with FDC non-susceptibility, and lineage 4 membership showed a marginal inverse association (OR 0.95, 95% CI 0.91–1.00, *p* = 0.042)—estimates based on 12 isolates (10 ST270) that we interpret cautiously given clonal confounding. Genotype-based prediction with sequence type-grouped five-fold cross-validation yielded AUC values of 0.775 (CT), 0.773 (CZA), 0.808 (FDC), and 0.749 (IMR).

## 3. Discussion

This multicenter genomic analysis of 442 CRPA isolates from eight Chinese provinces yielded three principal findings with direct implications for the design of molecular diagnostics and empiric treatment algorithms: carbapenemase carriage is uncommon (7.5%) and geographically heterogeneous, with 78% of CZA-non-susceptible isolates harboring no detectable carbapenemase gene; a non-carbapenemase co-selection score integrating eight non-carbapenemase resistance markers independently predicts MICs, with the strongest associations observed for CT (Spearman *ρ* = 0.639) and FDC (*ρ* = 0.586, largely attributable to ST270); and FDC retains near-universal activity across all molecular subgroups, although ST270-linked MIC elevation warrants ongoing surveillance. Together, these findings challenge the prevailing carbapenemase-centric paradigm for guiding novel agents’ therapy and highlight the clinical importance of chromosomal resistance mechanisms in CRPA.

The 7.5% carbapenemase carriage rate observed in this study is among the lowest reported in Chinese CRPA surveillance. In a national collection of 1168 CRPA isolates, Li et al. reported that ST463 alone—a KPC-2-producing clone concentrated in Zhejiang and Jiangsu provinces—accounted for 9.8% of strains; when combined with other carbapenemase-carrying sequence types, including ST235, the overall carbapenemase prevalence in that collection substantially exceeded the rate observed here [11]. In Fujian province, Xie et al. detected carbapenemase activity through phenotypic testing in 22.9% of CRPA, predominantly MBLs, with 68.8% of carbapenemase producers co-harboring two or more carbapenemase genes [18]. In Shanghai, Zhu et al. reported that ST270 was the most prevalent sequence type among 40 CRPA isolates (25%), with only a minority carrying acquired carbapenemase genes such as *bla*_GES-5_, while all 40 isolates harbored OprD mutations and 55% overexpressed the MexXY efflux pump—indicating that chromosomal mechanisms were the dominant resistance drivers even in a cohort where ST270 was already the predominant clone [19]. These comparisons underscore three important points. First, carbapenemase prevalence in Chinese CRPA varies considerably by geography: our finding of 7.5% in northern China contrasts with the 22.9% carbapenemase positivity rate reported in Fujian province and the higher rates observed in eastern China where carbapenemase-producing clones such as ST463 and ST235 are endemic. Second, the complete absence of ST463 from our collection—a clone that accounts for 76.3% of CZA-resistant CRPA in Hangzhou and carries a characteristic dual-effector genotype (*exoU*^+^/*exoS^+^*) linked to hypervirulence [12,13]—demonstrates that the dominant resistance mechanisms in one Chinese region may be absent from another, arguing against the extrapolation of molecular epidemiology findings across provincial boundaries when constructing national treatment guidelines. Third, the predominance of ST270, a clone that was recently described as harboring a purely chromosomally mediated carbapenem resistance repertoire (OprD inactivation, MexXY overexpression, and AmpC hyperproduction via AmpD/AmiD/DacC/DacB pathway disruption) [19], suggests that regional clonal expansion rather than nationwide dissemination of high-risk carbapenemase-producing clones shapes the CRPA landscape in Beijing and neighboring northern provinces.

ST270 was the dominant clone in this study, accounting for 30.8% (136 of 442) of all isolates and 64.6% (64 of 99) of carbapenemase-negative CZA-non-susceptible strains. This clonal concentration has been independently documented: in Zhu et al.’s Shanghai study, ST270 was the most prevalent ST (25%, 10 of 40 CRPA), with all ST270 isolates carrying OprD-inactivating mutations [19]; in a 2025 report from Zhejiang, a chromosomally integrated *bla*_KPC-87_ gene—located on a Tn3-based NTEKPC-IIa transposon with an inserted promoter element driving elevated expression—conferred CZA resistance in ST270 strains that caused a small-scale nosocomial outbreak followed by cross-regional dissemination upon patient transfer, demonstrating that ST270 can also serve as a platform for the chromosomal capture of carbapenemase genes under sustained antimicrobial pressure [20]. The identification of ST270 as the dominant chromosomal resistance clone across multiple independent Chinese studies, combined with its predominance in our northern China collection and the 2024–2025 emergence of FDC-non-susceptible subpopulations within this lineage (7.4% cumulative rate by 2025), raises concern that ST270 may be evolving toward a high-risk clone analogous to ST235 or ST244, albeit through a mechanistically distinct route—incremental accumulation of chromosomal resistance determinants rather than plasmid-borne carbapenemase acquisition. Whether the apparent geographic restriction of ST270 to China reflects incomplete global surveillance or true regional endemism remains to be determined.

The MBL versus serine–carbapenemase dichotomy was faithfully reproduced: all 28 MBL producers were CZA- and IMR-non-susceptible, while all five KPC-2 or GES-5/14 producers remained CZA-susceptible but were nonetheless IMR- and CT-non-susceptible, consistent with OprD porin deficiency compromising imipenem-based and ceftolozane-based activity, and/or with increased *bla*_KPC-2_ copy number and expression overwhelming the inhibitor as reported for ST463 [11], neither mechanism being directly measured in these two isolates. These findings are mechanistically predictable from the inhibitor spectra of avibactam and relebactam—diazabicyclooctanes that acylate the catalytic serine of class A and C β-lactamases but are inert against the zinc-dependent mechanism of MBLs [5]—and align with SENTRY surveillance data (<6% BL/BLI susceptibility among MBL-producing DTR *P. aeruginosa*) [8]. IDSA 2024 guidance, which designates FDC as the preferred agent for MBL-producing *P. aeruginosa* infections [9], is strongly supported: FDC retained 100% susceptibility among all 33 carbapenemase-positive isolates. However, the clinical utility of carbapenemase-guided therapy selection is constrained by the low pre-test probability of carbapenemase carriage. In our population, even a perfectly sensitive and specific rapid carbapenemase detection assay would directly inform treatment selection for only 7.5% of patients. For the remaining 92.5%, the key question is as follows: in a carbapenemase-negative CRPA isolate, which agent is most likely to be active?

The co-selection score was independently associated with BL/BLI non-susceptibility for CZA (aOR 1.60 per unit), IMR (aOR 2.00), and CT (aOR 2.58), with the strongest MIC correlation observed for CT (*ρ* = 0.639); the FDC association (*ρ* = 0.586) was substantially attenuated after excluding ST270 (*ρ* = 0.273), indicating clonal confounding. The eight genes—aminoglycoside-modifying enzymes (*aac(3)-IId*, *aac(6′)-Ib9*, *ant(2″)-Ia, aph(3″)-Ib, aph(3′)-Ia*, *aph(6)-Id*), *sul1*, and *toprJ3*—are markers of class 1 integron and genomic island carriage in *P. aeruginosa*; several are frequently carried on mobile genetic elements, so the score reflects cumulative non-carbapenemase resistance gene burden rather than strictly chromosomal events. In addition, these genes frequently co-localize with chromosomal AmpC (*bla*_PDC_) variants and efflux pump regulators on integrative conjugative elements [4,6]. The cumulative burden of these markers therefore reflects, albeit indirectly, the extent of genomic remodeling that accompanies the stepwise acquisition of high-level β-lactam resistance through the additive or synergistic effects of AmpC deregulation, OprD inactivation, and multidrug efflux pump overexpression.

The dominance of chromosomal over acquired resistance mechanisms is consistent with the emerging understanding that *P. aeruginosa* resistance to novel β-lactams is driven primarily by the mutational resistome. Oliver et al. recently catalogued the repertoire of chromosomal mutations that compromise newer β-lactams: AmpC Ω-loop mutations (e.g., E247K, G183D, and ΔG229–E247) confer high-level CT and CZA resistance while paradoxically producing collateral susceptibility to imipenem [5,21]; MexAB-OprM structural mutations enhance extrusion of both β-lactam substrates and diazabicyclooctane inhibitors; PBP3 (*ftsI*) mutations reduce β-lactam target binding; and large chromosomal deletions confer pleiotropic resistance through two-component regulator activation (ParRS, CpxRS) [5]. The González-Pinto et al. study of 61 isogenic PAO1 derivatives further demonstrated that combined NDM-1 production, MexAB-OprM overexpression, and OprD deficiency resulted in resistance to nearly all tested agents, including FDC [6]. Our non-carbapenemase co-selection score is a pragmatic proxy for cumulative non-carbapenemase resistance burden, though not as a mechanistic model—direct quantification of AmpC activity, porin loss, and efflux pump expression, combined with targeted Ω-loop sequencing, would be required to establish causal genotype–phenotype relationships.

FDC retained 100% susceptibility among carbapenemase-positive isolates and 97.3% overall, consistent with SENTRY data (98.1% FDC susceptibility among DTR *P. aeruginosa*) [8]. A recent study from Taiwan further confirmed >90% susceptibility across all imipenem-non-susceptible Gram-negative species, outperforming meropenem–xeruborbactam, cefepime–taniborbactam, and aztreonam–avibactam, for which activity was species-dependent [22]. FDC’s resilience—siderophore-mediated uptake through TonB-dependent receptors (PiuA, PirA, FptA) bypasses porin loss, combined with Ambler classes stability [6]—is well-characterized. However, the 12 FDC-non-susceptible isolates (10 ST270) merit scrutiny. Egge et al. demonstrated that loss-of-function mutations in TonB-dependent receptor genes (*pirR*, *pirS*, *pirA*, *piuA*, *piuD*) are present in up to 25% of clinical *P. aeruginosa* populations without prior FDC exposure and that isolates harboring major TBDR mutations are enriched for the FDC heteroresistance phenotype [23]. These pre-existing variants lead to the outgrowth of fully resistant subpopulations during FDC therapy, as documented in a clinical case of stepwise evolution of heteroresistance to frank resistance during prolonged monotherapy [24]. Kang et al. demonstrated that FptA inactivation—the most common first-step FDC resistance mutation—impairs *P. aeruginosa* growth under iron-depleted conditions through disruption of siderophore-mediated iron homeostasis, establishing a fitness cost that may constrain the dissemination of FDC-resistant clones [25]. The clinical relevance of FDC heteroresistance extends beyond *P. aeruginosa*: Warecki et al. recently highlighted its emergence in NDM-producing *Enterobacterales* and cautioned that standard susceptibility testing methods may fail to detect subpopulations capable of outgrowth during FDC therapy [26]. The temporal MIC drift in ST270 (*ρ* = 0.22, *p* = 0.009) and the 2024–2025 emergence of FDC-non-susceptible ST270 isolates are consistent with temporal emergence through TBDR pathway attrition. The enrichment of ST270 among FDC-non-susceptible isolates is confounded by single-center sampling and does not constitute evidence of nationwide resistance. Nevertheless, that all 12 FDC-non-susceptible strains were carbapenemase-negative reinforces the central argument: chromosomal mechanisms dominate the resistance landscape.

The inverse association between ExoU carriage and MICs—approximately two-fold lower for FDC and four-fold lower for CT—is a novel observation. The *exoU* gene encodes a phospholipase A_2_ cytotoxin and marks globally disseminated high-risk clones, including ST235 and ST357 [4,27]. In the POP cohort, *exoU*-positive strains were independently associated with higher 30-day mortality [2]. The paradoxical finding that the most cytotoxic strains are simultaneously the most susceptible has not, to our knowledge, been systematically reported. Multivariable regression confirmed that *exoU* positivity was independently protective against CZA non-susceptibility (aOR 0.34, 95% CI 0.14–0.83, *p* = 0.018) after adjustment. Three mechanisms may explain this: a biological association (metabolic burden of PAPI-2 maintenance under antibiotic selection, supported by the negative virulence–resistance gene score correlation and the FptA fitness cost precedent [25]; clonal confounding (*exoU*-positive STs are intrinsically more susceptible, *exoU*-negative ST270 is heavily drug-resistant); and the emergence of dual-positive ST463 strains demonstrating that the relationship can be inverted under strong selection [12,13].

The CACTUS trial demonstrated that CT was associated with higher clinical success than CZA for MDR *P. aeruginosa* pneumonia (63% vs. 51%, aOR 2.34), though treatment-emergent resistance occurred at 22–23% with both agents [28]. The GAME CHANGER trial—the first RCT of FDC for carbapenem-resistant Gram-negative bloodstream infections—confirmed FDC non-inferiority to the standard of care for 14-day mortality (8% vs. 7%; risk difference 1%, 95% CI −3 to 6), including the carbapenem-resistant subset [29]. These outcome data underscore the stakes of correct empiric selection, yet current molecular diagnostics are fundamentally unable to detect the chromosomal resistance mechanisms driving most non-susceptibility [7]. In a recent evaluation, 76% of CRPA tested negative based on NG-Test CARBA 5, and 52% were negative even based on PCR [17].

A pragmatic two-tier approach emerges. First, rapid carbapenemase gene detection (∼1 h) identifies the minority with MBL, KPC, or GES: MBL-positive infections should receive FDC; KPC- or GES-positive infections can be treated with CZA or FDC [9]. Second, for the carbapenemase-negative majority (92.5%), direct phenotypic susceptibility testing remains the only reliable strategy. FDC provides the broadest empiric coverage pending results, with the caveat that ST270-predominant settings may harbor a small FDC-non-susceptible subpopulation. The non-carbapenemase co-selection score and *exoU* status are candidate markers for future expanded molecular panels, but their translation requires assay development and prospective validation.

Several limitations warrant consideration. First, the collection is dominated by a single center (86.4% of isolates) and consists entirely of respiratory specimens; these findings reflect a specific subset of CRPA in China and should not be interpreted as nationally representative. Second, the cross-sectional design precludes causal inference about temporal MIC trends; the longitudinal data within ST270 require prospective confirmation with paired clinical outcome data. Third, resistance gene detection through whole-genome sequencing does not measure transcript or protein expression; AmpC hyperexpression and OprD loss were inferred from gene disruption signatures and may not fully capture functional resistance status. Fourth, the MIC testing range (upper limit > 64 mg/L) created a detection ceiling that may underestimate resistance magnitude in highly resistant subpopulations. Fifth, the co-selection score is a correlative metric developed post hoc; individual gene contributions were not validated through gene knockout or complementation, several constituent genes are integron- or plasmid-associated, and the score’s generalizability to other geographic settings has not been established.

Chromosomal resistance mechanisms—not acquired carbapenemases—dominate non-susceptibility in this CRPA collection. Carbapenemase-centered molecular testing misses 78% of CZA resistance. FDC retains the broadest coverage across all molecular subgroups and should be the empiric agent of choice, though clonal MIC drift warrants ongoing surveillance. The ExoU virulence–resistance association suggests that the most cytotoxic strains may be paradoxically the most treatable. China’s CRPA molecular epidemiology is geographically heterogeneous—national guidelines should not extrapolate regional resistance mechanism distributions without local surveillance data.

## 4. Materials and Methods

### 4.1. Bacterial Isolates

We collected 442 clinical *P. aeruginosa* isolates from 10 hospitals across eight Chinese provinces between 2017 and 2025. The participating provinces were Beijing (3 hospitals), Heilongjiang, Guangdong, Ningxia, Jiangxi, Shandong, Hubei, and Hebei. Only one isolate per patient was included. Species identification was performed at each participating center using matrix-assisted laser desorption ionization–time of flight mass spectrometry (MALDI-TOF MS; Bruker Daltonics, Bremen, Germany) and confirmed by the central laboratory. Isolates were stored at −80 °C in glycerol broth until testing.

### 4.2. Antimicrobial Susceptibility Testing

MICs for all collected isolates were determined in the central laboratory through broth microdilution using 96-well panels (BIO-KONT, Wenzhou, China) in cation-adjusted Mueller–Hinton II broth (BD BBL, Sparks, MD, USA); for FDC, the broth was rendered iron-depleted in accordance with the CLSI M100 (2025) recommendations for FDC susceptibility testing. Imipenem, meropenem, and four novel agents were tested: FDC, CZA (avibactam fixed at 4 mg/L), IMR (relebactam fixed at 4 mg/L), and CT (tazobactam fixed at 4 mg/L). The MIC testing range was 0.032 to 64 mg/L for all six drugs. MICs exceeding the upper limit of the testing range were recorded as above the highest concentration tested and reported as >64 mg/L. *P. aeruginosa* ATCC 27853 was used as the quality control strain. Susceptibility was interpreted according to Clinical and Laboratory Standards Institute (CLSI) 2025 clinical breakpoints.

### 4.3. Whole-Genome Sequencing and Resistance Gene Detection

Genomic DNA was extracted from overnight cultures using the STE method. DNA libraries were prepared and sequenced on the Illumina NovaSeq X plus platform (Illumina, San Diego, CA, USA) generating 150 bp paired-end reads. Raw reads were quality-filtered using fastp v0.23.1. The mean sequencing depth was 241× (range, 117–567×); assembled genomes comprised a median of 138 contigs (N50, 334 kb; genome size, 6.85 Mb [range, 6.17–12.16 Mb]). Assemblies were screened for contamination through inspection of genome size, contig count, and N50; the eight genomes exceeding 1000 contigs or 7.8 Mb were individually reviewed, and resistance gene calls were verified against phenotypic susceptibility profiles—two of these carried *bla*_IMP-1_ or *bla*_IMP-45_/*bla*_VIM-1_ with concordant MBL phenotypes. No isolate was excluded. De novo assembly was performed using SPAdes v4.2.0 [30]. Acquired and chromosomal resistance determinants were identified with ABRicate v1.4.0 using the ResFinder and Comprehensive Antibiotic Resistance Database (CARD) databases. We used a minimum identity threshold of 80% and minimum coverage of 80%. Virulence factor genes were identified with ABRicate against the Virulence Factor Database (VFDB). The last update date of the above database was 3 April 2026. Multilocus sequence typing (MLST) was performed with mlst v2.33.1 using the PubMLST *P. aeruginosa* scheme.

For each gene, we recorded presence or absence as a binary call. Genes detected by both ResFinder and CARD under different naming conventions (e.g., *bla*_IMP-45_ from ResFinder and IMP-45 from CARD) were merged into a single detection signal. Where the two databases disagreed, we used a union approach: a gene was considered present if either database detected it.

### 4.4. Carbapenemase Classification

Carbapenemase genes were classified into three groups based on Ambler molecular class and the clinical activity of avibactam. Metallo-β-lactamase (MBL) genes included *bla*_IMP-1_, *bla*_IMP-45_, *bla*_IMP-104_, *bla*_VIM-1_, *bla*_VIM-2_, and *bla*_DIM-1_ (Ambler class B, not inhibited by avibactam). Class A carbapenemases susceptible to avibactam inhibition were further divided into two subgroups: the KPC group (*bla*_KPC-2_) and the GES group (*bla*_GES-5_ and *bla*_GES-14_). Strains carrying no detectable carbapenemase gene were classified as carbapenemase-negative. Strains with genes from more than one group were classified as multiple.

### 4.5. Non-Carbapenemase Co-Selection Score

To quantify the cumulative burden of non-carbapenemase resistance, we screened all 221 detected resistance determinants (excluding the nine carbapenemase genes) using the following criteria: (i) prevalence between 5% and 95% in the full collection (the 5% lower bound excludes singleton/rare genes and carbapenemase genes, all of which had prevalence < 5%; the 95% upper bound excludes species core genes present in nearly all strains); and (ii) annotation as non-carbapenemase resistance determinants outside of the species core gene set; several of these genes are class 1 integron or genomic island markers in *P. aeruginosa* per the ResFinder and CARD database entries. These criteria were applied before any association testing with the phenotypes. Because short-read sequencing cannot always resolve whether a gene is chromosomal or plasmid-borne, this score is not strictly chromosomal. Eight genes satisfied both criteria: *aac(3)-IId*, *aac(6′)-Ib9*, *ant(2″)-Ia*, *aph(3″)-Ib*, *aph(3′)-Ia*, *aph(6)-Id*, *sul1*, and *toprJ3*. None of these genes are known to hydrolyze or directly confer resistance to any of the four agents tested; the score serves as a quantitative proxy for the cumulative burden of non-carbapenemase resistance adaptations—including AmpC (PDC) overexpression, OprD porin loss, and efflux pump upregulation—under sustained antimicrobial pressure.

### 4.6. Gene Selection for Specific Analyses

Different subsets of resistance determinants were used for different analyses. For the resistance gene landscape heatmap, carbapenemase genes were included regardless of their prevalence; other genes required prevalence ≥ 3 strains and ≤95%, yielding 89 genes in three categories: carbapenemase (*n* = 9), other β-lactamase (*n* = 44), and other resistance genes (*n* = 36). For the volcano plot of CZA non-susceptibility correlates, all 221 resistance determinants were screened within the carbapenemase-negative subset (*n* = 409). For the molecular portrait of FDC non-susceptible strains, genes were selected if they were carbapenemase genes, had ≥2 occurrences among the 12 FDC non-susceptible strains and overall prevalence below 95%, or carried established biological relevance (*bla*_PER-1_), producing 22 genes. For genotype-based prediction, we used all resistance genes with 2–95% prevalence (*n* = 50), all nine carbapenemase genes, and the non-carbapenemase co-selection score, yielding 60 features. The virulence gene score was computed as the sum of all 136 VFDB-detected virulence factors. The T3SS effector genotype (*exoU*, *exoS*) was extracted from the VFDB output and classified as cytotoxic (*exoU*^+^/*exoS*^−^), invasive (*exoU*^−^/*exoS*^+^), dual-positive, or dual-negative.

### 4.7. Statistical Analysis

All statistical analyses were performed in Python 3.9.2. MIC distributions between two independent groups were compared with the Mann–Whitney *U* test; comparisons involving more than two groups used the Kruskal–Wallis test. For raincloud plots, hypothesis testing was restricted to MBL versus carbapenemase-negative strains. Correlations between continuous variables were assessed with Spearman’s rank correlation coefficient (*ρ*). Categorical associations were tested with Fisher’s exact test (2 × 2 tables) or the chi-square test (larger contingency tables). For the volcano plot, FC of gene prevalence between CZA-non-susceptible and CZA-susceptible strains within the carbapenemase-negative subset was computed; *p*-values were obtained through Fisher’s exact test and transformed to −log_10_ scale. Genes with an FC > 2 and a Benjamini–Hochberg false discovery rate-adjusted *p* < 0.05 were considered significantly enriched.

Independent predictors of non-susceptibility were identified through multivariable logistic regression. Predictors included carbapenemase carriage, non-carbapenemase co-selection score, ExoU status, ST270 membership, age (centered at the cohort median, 67 years), gender, hospital (dominant center vs. others), province (Beijing vs. others), isolation year, and clonal lineage (four major ST clusters vs. others). Results are reported as adjusted odds ratios with 95% confidence intervals.

For genotype-based prediction, 58 features were entered into a logistic regression classifier with L2 regularization (C = 0.1, class_weight = ‘balanced’) evaluated by 5-fold cross-validation grouped by sequence type so that isolates of the same ST never spanned training and test folds. Model discrimination was assessed by the cross-validated area under the receiver operating characteristic curve (AUC). Confidence intervals for proportions were calculated using the Wilson score method. All tests were two-sided; *p* < 0.05 was considered statistically significant. Gene-level enrichment testing was corrected for multiple testing using the Benjamini–Hochberg method.

## 5. Conclusions

Non-carbapenemase resistance determinants were strongly associated with the non-susceptibility of these new agents, although direct causal contributions were not measured. Carbapenemase-centered molecular testing misses 78% of CZA resistance. FDC retains the broadest in vitro coverage across all molecular subgroups and is the preferred agent for carbapenemase-producing strains; whether it should be the empiric agent of choice requires confirmation in studies with clinical outcome data, and clonal MIC drift warrants ongoing surveillance. The ExoU virulence–resistance association suggests that the most cytotoxic strains may be paradoxically the most treatable. China’s CRPA molecular epidemiology is geographically heterogeneous—national guidelines should not extrapolate regional resistance mechanism distributions without local surveillance data.

## Data Availability

The original data presented in the study are openly available in GenBank Database with BioProject ID PRJNA1519060.

## Supplementary Materials

The following supporting information can be downloaded at https://www.mdpi.com/article/10.3390/antibiotics15090835/s1, Table S1: Epidemiological data, resistance genes, and virulence genes of all CRPA isolates.

## Abbreviations

AUC	Area under the receiver operating characteristic curve
BL/BLI	β-lactam/β-lactamase inhibitor
CARD	Comprehensive Antibiotic Resistance Database
CLSI	Clinical and Laboratory Standards Institute
CRPA	Carbapenem-resistant *Pseudomonas aeruginosa*
CT	Ceftolozane–tazobactam
CZA	Ceftazidime–avibactam
DTR	Difficult-to-treat resistance
ECDF	Empirical cumulative distribution function
FDC	Cefiderocol
FC	Fold change
IMR	Imipenem–relebactam
MBL	Metallo-β-lactamase
MALDI-TOF MS	Matrix-assisted laser desorption ionization–time of flight mass spectrometry
MIC	Minimum inhibitory concentration
MLST	Multilocus sequence typing
OR	Odds ratio
ROC	Receiver operating characteristic
ST	Sequence type
T3SS	Type III secretion system
TBDR	TonB-dependent receptor
VFDB	Virulence Factor Database
WHO	World Health Organization
